# Roe Deer (*Capreolus capreolus*) Hair as a Bioindicator for the Environmental Presence of Toxic and Trace Elements

**DOI:** 10.3390/toxics11010049

**Published:** 2023-01-03

**Authors:** Susanna Draghi, Stella Agradi, Federica Riva, Duygu Tarhan, Bengü Bilgiç, Banu Dokuzeylül, Alev Meltem Ercan, Mehmet Erman Or, Gabriele Brecchia, Daniele Vigo, Francesco Arioli, Federica Di Cesare, Giulio Curone

**Affiliations:** 1 Department of Veterinary Medicine and Animal Sciences (DIVAS), University of Milan, Via dell’Università 6, 26900 Lodi, Italy; 2 Biophysics Department, Cerrahpasa Faculty of Medicine, Istanbul University-Cerrahpasa, Topkapı, Turgut Ozal Millet Cd, Fatih, 34093 Istanbul, Turkey; 3 Internal Medicine Department, Faculty of Veterinary Medicine, Istanbul University-Cerrahpasa, Istanbul Universitesi Avcılar Kampusu, Baglariçi Cd. No:7, Avcılar, 34320 Istanbul, Turkey

**Keywords:** bioindicators, wild animals, ecotoxicology, trace elements, potentially toxic elements

## Abstract

The return to pasture use as an alternative to intensive livestock farming implies some risks with the lack or the excessive presence of potentially toxic elements; in this regard, wild animals have been used as bioindicators for decades. Thus, the purpose of this study is quantifying Cu, Cr, Mn, Zn, Se, As, Cd, Ni, Pb, Al, Fe, and Mg in fur from roe deer and understanding if it is a valid bioindicator tool. Hair was collected from 39 hunted roe deer and divided by age (<36 months old/≥36 months old), sex (male/female), and area of origin (urbanized/rural area). The mean concentrations of Fe, Mg, Mn, Al, Cr, and Pb were higher (*p* < 0.05) in the urbanized group; the mean levels of Mg and Cr were higher (*p* < 0.05) in older animals; and Cu, Fe, Mg, Cd, and Cr showed a higher accumulation in females. Our findings showed an age-related variation of elements, with higher concentrations in adult animals and females. In conclusion, our findings prove that hair is a valid matrix for this type of survey, and wild animals are good bioindicators for monitoring the presence of trace elements in pastures.

## 1. Introduction

Nowadays, a lot of global challenges are affecting agricultural and zootechnical productions that, in this context, need to be more environmentally friendly and sustainable. Such challenges are population increase, urbanization with the increase in environmental degradation, the reduction of available land for agriculture, and most importantly: climate change [1]. In the last few decades, environmental issues have been the first major driver for change in agriculture management. For example, production strategies were prompted to reduce water and energy consumption. Moreover, the sustainability of food production, in particular, food of animal origin, has been a big challenge because it is strictly linked to the competition between humans and zootechnical animals for food sources [2,3]. Furthermore, after two years of the pandemic and the outbreak of war in Ukraine, the challenges for agriculture and animal breeding have suddenly changed again because of the dramatic increase in raw materials’ prices, especially cereals, putting intensive farming in crisis. In this context, intensive farming is becoming unstainable from environmental and economical points of view, imposing a reflection on more sustainable production processes, such as the exploitation of pastures [4,5,6]. One of the challenges encountered in moving to the major exploitation of pastures could be the imbalance of Potentially Toxic Elements (PTEs). Indeed, elements such as copper (Cu), manganese (Mn), magnesium (Mg), iron (Fe), selenium (Se), nickel (Ni), chromium (Cr), cadmium (Cd), lead (Pb), zinc (Zn), aluminium (Al), arsenic (As), and several others are widespread in the environment and are classified as essential or non-essential based on their functions. Essential elements include, for example, Cu, Mn, Se, Ni, Cr, and Zn. They are essential because are necessary to several physiological functions: Se, Zn, and Cu are all involved in immune functions; Se is necessary for growth and fertility and is an important enzymatic cofactor; copper and zinc were used in pigs and poultry as growth promoters; nickel activates the enzyme involved in haematopoiesis and affects iron metabolism; chromium is required for normal carbohydrate metabolism, and iron is present in several enzymes, such as ferritin, responsible for electron transport and the activation and transport of oxygen [7,8]. Aluminium and arsenicum are generally not essential for life, and being reactive, they have the potential to interact and influence biochemical pathways and cellular processes [9,10]. Chromium is considered a two-faced element because it has biological functions, but when it exceeds safe concentrations, it can cause a variety of clinical problems. Cr is also considered an important bio-element because it has an exceptional role in metabolic processes [11]. On the other hand, there are non-essential elements that are classified as toxic. Two main examples are cadmium and lead, which are tolerated by biota only at low concentrations and thus considered toxic [12,13]. Cd does not own physiological functions in organisms. It is a teratogen, carcinogen, and mutagen element. It can cause a reduction in growth, and these effects are also exerted at low concentrations [14]. Despite the division into essential and non-essential, or toxic and trace, all these elements are considered PTEs. Indeed, even essential elements in concentrations above safe levels can cause adverse health effects; this property has been defined as hormesis [15,16]. Although plants are often used as biomonitors, they only indicate the burden of environmental pollution over a certain timespan; when both temporal and spatial information are required, animals can be used [17]. As reported by several authors, the animal organism selected for biomonitoring should follow several criteria: First, the spatial representativeness needs to be considered, as the species should be widespread in the studied area. Secondly, the practicability of specimen collection needs to be considered; many species are regularly hunted, which means that no additional killing is necessary Finally, at least the physiological characteristics, feeding habits, and annual and daily rhythms of the species must be known [15,17]. Following these criteria, one of the species that can be used for ecotoxicology studies and biomonitoring of small areas is the roe deer. The roe deer species is widely distributed across the world, showing a large behavioural plasticity that makes these animals adaptable to a wide range of habitats [18]. Despite the wide geographic distribution of the species, individual roe deer live in small home ranges between 16 and 80 ha and thus, the killing area could be considered representative of the home range of the individual [19]. Moreover, the wild roe deer’s diet consists mainly of plants, roots, and minerals obtained from the soil through licking. For the biomonitoring of environmental pollution in wild animals, a lot of non-invasive collection methods of samples such as hair (fur) and feathers have been proposed and investigated. Fur resulted a good matrix for the assessment of the long-term exposure of the organism to toxic or trace elements [16]. Indeed, the concentration of contaminants in fur reflects the exposure during the period in which the hair is growing; several studies have shown that the hair of animals reflects the accumulation and concentration of elements from the previous months and years [20]. It has also been suggested that the metals’ absorption ability could vary in relation to several aspects, such as different hair morphology in different mammalian species. Hair is an epidermal appendage composed of three layers: the innermost layer called the medulla, composed of columns of keratinized cells; the middle layer called the cortex, in which pigment granules are contained; and the most external layer called the cuticle, composed of plates of cells characteristic for each species [21,22]. The differences between the three layers confer different affinities for compounds; for example, the cuticle contains sulphur, which attributes to hair the ability to absorb metals [23]. Meanwhile, the medulla and the cortex contain little or no sulphur, but both layers are embedded in pigment granules that confer the ability to selectively bind metal elements [21,22]. Due to these peculiar features, fur is a more reliable method for monitoring the trace and toxic elements’ status in the body than blood or urine, which are often affected by the temporary influences of some factors and are not matrices of accumulation [24]. Therefore, hair has been individuated as one of the most important matrices for biological monitoring in the Global Environmental Monitoring System (GEMS) [25]. Based on our knowledge, there are still few studies on the content of elements in the fur of wild animals, and no basal levels are reported for PTEs in wild species. Usually, muscle, liver, or kidney are used. Moreover, the detection of environmental pollutants in soil, grass, and water may not be a sufficient indication of risk definition in the use of a pasture because the bioaccumulation depends on the physiological features of the single species, the gender, and the age of animals [26], but may also depend on other factors such as species-specific morphology. For example, ruminants may reduce toxicity through ruminal bacterial digestion or increase it through ruminal metabolism [27,28,29].

Thus, taking into account the factors influencing bioaccumulation and the possible sharing of the pastures between wild and livestock animals, the objective of this study is the assessment of the toxic elements (Al, As, Cd, Cr, Ni, and Pb) and the trace elements (Cu, Fe, Mg, Mn, Se, and Zn) in roe deer hair from two different environmental areas of northern Italy in order to evaluate if the fur is a useful matrix that reflects the different home ranges, and consequently, if it is possible to determine the healthiness of the pasture using the roe deer fur as a bioindicator tool.

## 2. Materials and Methods

### 2.1. Sample Collection and Georeferencing

**Figure 1:**
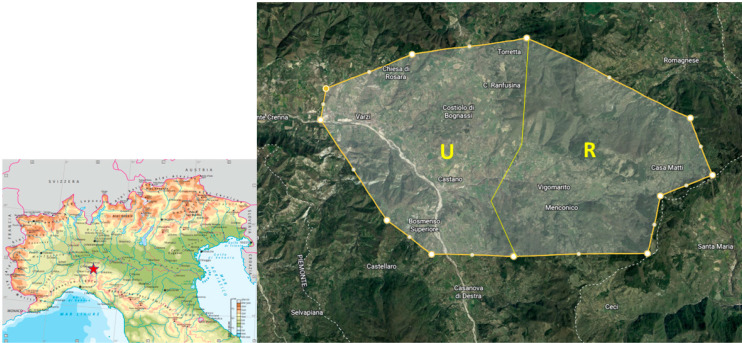
Map of the sampling area. U: Urbanized area; R: Rural area.

The fur was collected from 39 hunted roe deer (Capreolus capreolus): 17 females and 22 males. Based on age, animals were divided into two groups: younger than 36 months (17 animals; 6 females and 11 males) and older than 36 months (22 animals; 11 females and 11 males). Animals were killed by licensed people during the hunting season of 2021. According with the hunting plan, samples from females were collected during June, November, and December, while samples from males were collected in June, August, and September. All the sampling procedures were performed at hunting meat processing plants and after the slaughter of the animals during the regular hunting activity by authorized hunters. In order to reduce the variability among animals, hair samples were collected behind the arcus costalis on the left side of the animals. Hairs were cut using an electric shaver, close to the skin and from a 10 × 10 cm area, then stored in polyethylene bags in a dry and dark place until further analysis. The animals enrolled in the study came from a specific area of northern Italy of about 5500 ha (Figure 1). During the sampling procedures, the georeferentiation of the killing area was registered through the use of a Garmin GPSMAP^®^ 65. Animals were further grouped based on the hunting area where they were killed. In particular, two areas were categorized based on the amount of anthropogenic activity and urbanization: the rural area (20 animals; 7 females and 13 males) and the urbanized area (19 animals; 10 females and 9 males). This study complied with Italian and International laws on animal experimentation and ethics (Animal Welfare Organisation of Milan University; the number of authorization n°26_2022).

### 2.2. Trace and Toxic Elements Analysis

Chromium (Cr), copper (Cu), iron (Fe), magnesium (Mg), manganese (Mn), selenium (Se), zinc (Zn), arsenic (As), nickel (Ni), lead (Pb), cadmium (Cd), and aluminium (Al) elements were analysed by using an inductively coupled plasma-optical emission spectrophotometer (ICP-OES; Thermo iCAP 6000 series) at the Trace and Toxic Element Analysis Laboratory of the Biophysics Department, Cerrahpaşa Faculty of Medicine, Istanbul University-Cerrahpaşa. The method of wet decomposition for trace and toxic element measurements was used. The average sample weight used was 0.04 g. According to this method, all samples were dissolved in a drying oven (Heraeus W.C., Hanau, Germany) at 180 °C by adding 2 mL of 65% nitric acid (Merck, Darmstadt, Germany) and 1 mL of 60% perchloric acid (Panreac, Barcellona, Spain). The suspension was vortexed after being cooled at room temperature, and distilled water was added to the samples at 10 mL total volume. The evaluation of the concentration of each element was made one by one according to the weight of the sample. Each sample analysis was repeated three times by the device and averaged for results. Trace and toxic elements levels were expressed as µg·g^−1^ sample wet weight. ICP-OES device parameters for the determination of trace and toxic elements are summarized in Table 1. Standard solutions of each element were prepared by using solutions containing 1000 ppm (mg/L) in deionized water of each tested element (Chem-Lab NV, Zedelgem, Belgium). Distilled water was used as the blank solution. Reproducible and linear calibration curves were obtained by using standard solutions and blank solutions. Thus, the correlation coefficient of the calibration curve was found for each studied element. In the study, the appropriate wavelengths (Table 2) of all elements were used for the analysis by the ICP-OES device.

### 2.3. Statistical Analysis

The statistical analysis was performed through the use of the software GraphPad InStat 3^®^. The comparison between hair toxic element concentrations and age, sex, and geographic area was evaluated by the use of the unpaired *t*-test with Welch correction or Mann–Whitney Test in the cases of normally and non-normally distributed data, respectively. Age, sex, and geographic areas were categorized into two different levels. The differences between groups (Group A = aged < 36 months.; Group B = aged ≥ 36 months; male/female; urbanized/rural) were considered statistically significant when the *p*-value was <0.05.

## 3. Results

### 3.1. Method Validation

Table 3 shows the results of method validation.

### 3.2. Average Concentrations of Trace and Toxic Elements

The mean concentrations of trace and toxic elements are presented in Table 4.

The average content of chromium identified in the hair of roe deer was 1.61 mg·kg^−1^ and the maximum level obtained was 6.58 mg·kg^−1^; in the case of copper, a mean content of 7.49 mg·kg^−1^ was detected; in the case of Mn, the average content was 3.10 mg·kg^−1^, while for Se it was mg·kg^−1^; and in the case of As, the average concentration found was 1.97 mg·kg^−1^, but the maximum level was 6.80 mg·kg^−1^ and no sample was quantified to zero. Cadmium’s mean concentration was 0.08 and the maximum level reached was 0.99 mg·kg^−1^. The mean concentrations registered for nickel and lead were 0.82 and 1.39 mg·kg^−1^, respectively, with a maximum level of 6.78 mg·kg^−1^ in the case of nickel and 9.42 mg·kg^−1^ in the case of lead. The average concentration registered for Al was 140 mg·kg^−1^ with a maximum level of 1800 mg·kg^−1^. In the case of iron, the mean level was 160 mg·kg^−1^; for Mg and Zn, the average concentrations were 230 and 70 mg·kg^−1^, respectively.

### 3.3. Average Concentration Comparison between the Rural and Urbanized Area

The comparison between the average concentrations of trace and toxic elements is shown in Table 5.

In this study, the mean concentration of iron in roe deer fur collected from animals belonging to the urbanized area was 160 mg·kg^−1^, significantly higher (*p* < 0.01) than the average concentration found in animals belonging to the rural area, which was 90 mg·kg^−1^. In the case of Mg, the average content in hair was significantly higher (*p* < 0.05) in animals belonging to the urbanized area (260 mg·kg^−1^) compared to those belonging to the rural area (200 mg·kg^−1^). The mean content of manganese registered in the urbanized area was 4.35 mg·kg^−1^, significantly higher (*p* < 0.01) than the mean level identified in the rural area (1.91 mg·kg^−1^). The average content of aluminium in the urbanized area was 220 mg·kg^−1^, while in the rural area was 70 mg·kg^−1^, thus was significantly higher in the urbanized area than in the rural area (*p* < 0.05). The average content of chromium was significantly higher in the urbanized area than in the rural area (*p* < 0.001) with mean concentrations of 2.01 and 1.23 mg·kg^−1^, respectively. Moreover, for lead a significantly higher (*p* < 0.05) content in the fur of roe deer belonging to the urbanized area (1.99 mg·kg^−1^) than that registered in those belonging to the rural area (0.83 mg·kg^−1^). In relation to the other trace and toxic elements considered no statistically significant difference was found, but the concentrations observed in animals from the urbanized area were higher than in animals sampled in the rural area.

### 3.4. Average Concentration Comparison between Age Classes

The statistically significant differences between animals belonging to the class of age <36 months and those belonging to the class ≥36 months are presented in Table 6.

The average content of Mg was statistically significantly higher in older animals than in younger animals (*p* < 0.05), with average concentrations of 260 and 180 mg·kg^−1^, respectively. In the case of chromium, the mean content in the fur of older animals was 1.96 mg·kg^−1^, while in younger animals it was 1.17 mg·kg^−1^, showing a significantly higher content in the fur of older roe deer (*p* < 0.01). For the other elements considered in the study, no statically significant differences were detected, but all tended to be concentrated higher in older animals.

### 3.5. Average Concentration Comparison between Sexes

Differences in the average concentrations of trace and toxic elements identified between males and females are shown in Table 7.

The average content of copper measured in the hair of females was 8.23 mg·kg^−1^, while in males it was 6.85 mg·kg^−1^, resulting significantly higher in females (*p* < 0.05). The mean iron quantity detected was 160 mg·kg^−1^ and 150 mg·kg^−1^ for females and males, respectively; the content of iron was significantly higher in females (*p* < 0.05). Mg showed higher content in female fur than male fur (*p* < 0.05) with an average content of 280 mg·kg^−1^ and 180 mg·kg^−1^, respectively. Even in the case of cadmium, a significantly higher content was measured in the hair of females (*p* < 0.05); the mean content of cadmium in the hair of females was 0.09 mg·kg^−1^ while it was 0.07 mg·kg^−1^ in males. Chromium showed a significantly higher mean concentration in females (*p* < 0.001) with a content of 2.13 mg·kg^−1^. No other statistically significant differences were recorded between the two sexes. Except for arsenic and manganese, the other trace and toxic elements considered in this study tended to be higher in female animals.

## 4. Discussion

In general, no basal concentrations of trace and toxic elements in the hair or fur from wild animals are reported in the literature. The content of aluminium registered was comparable with levels measured in other studies and other species of both wild and livestock animals [27,28]. Aluminium is the third most abundant element in the earth’s crust, after oxygen and silicon; through the ingestion of contaminated food, aluminium enters the food chain, and in this way, it is bioaccumulated and biomagnified [9]. The presence of aluminium in the soil can result from both natural deposition and human activities [30]. Because of anthropogenic activities, the arable lands are acidic, and this causes the mobilization of aluminium from sediments, entering this way into terrestrial and aquatic food chains. Despite its abundance and high availability in the environment, it seems that it does not own biological functions in the organisms [31]. It has been demonstrated that it has a high affinity for hair, and there are several investigations about its levels in mammalian fur. In our study, the average concentration of Al detected was higher than the one reported by Kucharzak et al. in a study conducted on the kidneys, liver, and muscle from game animals [32]. These results are consistent with both the high affinity of aluminium for hair and the clay soils in the area from which the roe deer involved in the study were hunted; indeed, studies have demonstrated that clay soils are rich in aluminium, which takes part in soil stabilization. Moreover, at low pH, aluminium oxide precipitates on the surface of clay minerals, creating a coating and becoming available for the biota [33]. Arsenic is a metalloid, and it is regularly detected in the environment. Its occurrence in the environment is due to its presence in rocks and sediments and as a result of human industrial activities [34]. This element is rarely investigated in wild mammals, but the concentration we detected seems to be higher than those registered by Squadrone et al. in a study conducted on wild animals’ hair [26]; the divergences are probably due to the different sampling areas, which can be related to a difference in soil composition and thus to the presence of sediment that can release arsenic. Cadmium is a by-product of zinc and lead production; it has several other industrial applications such as in cadmium-nickel batteries, plastics, pigments, and ceramics, and it is able to accumulate in plants fertilized with cadmium-contaminated sewage [35]. In this study, an average concentration similar to those reported by other authors was registered. Differently, in a study conducted on fur from roe deer, wild boars, and hares from Poland, the maximum level detected was lower; the higher concentration of cadmium in the studied area was probably due to the presence of a galvanised sheet metal production company [32]. Chromium originated mainly from anthropogenic sources such as industrial and combustion processes. The average concentration detected in fur was in accordance with previous studies on wild animals and dairy cows [34,35]. Copper is an essential element, and it enters a lot of physiological functions, being a part of different enzymes and proteins. For example, copper is required for terminal oxidation and the elimination of free radicals and is involved in the synthesis of hormones and iron metabolism [36]. Both copper deficiency and poisoning, especially in ruminants, are responsible for nutritional and nutrition-related diseases and, in the case of livestock, for economic losses due to the mortality of animals. The mean Cu concentration we found in roe deer hair was higher than those reported in cows and silver foxes [37,38]; the differences may be explained by both the different physiological aspects of the species examined and the different sampling areas. Iron is the fourth most abundant metal in the earth’s crust and is the essential metal with the highest concentration in all species. As an essential trace element, it participates in several metabolic reactions and it is a component of haemoglobin, myoglobin, and several other proteins, with an essential role in the transport and utilization of oxygen [7]. The mean concentration of Fe in hair samples was similar to values observed in another study in red deer from the Central Alps [39]. The average concentration of lead found in this investigation was lower than the quantity registered in previous studies conducted on the same species in Poland; these differences could be due to the different levels of environmental contamination. Indeed, in the past, lead was widely used, for example, in paints, lubricants, pesticides, and industry; thus, the higher or lower presence of anthropogenic activities could influence the quantity of Pb present in the environment and in the tissues of living organisms [26]. Due to its multiple applications, lead has become a ubiquitous environmental pollutant in air, water, soil, and food [40]. It is widely recognized that lead is highly toxic for both animals and humans, and for this reason, it is considered one of the most dangerous minerals to health. Lead is poorly absorbed and scarcely excreted only through bile and urine. Toxicokinetic studies showed that similarly to cadmium, it can bioaccumulate in the hair at high concentrations; thus, it could be used for monitoring environmental pollution using animals as sentinels [41]. Magnesium is required by all living organisms. Deficiencies are uncommon but documented; one example is ruminant hypomagnesemia, also known as ruminant grass tetany [8]. The average content of manganese in roe deer hair is lower than those registered in wild red deer and alpaca reared in a semi-extensive way in north-western Italy [27,28]. Mn is an essential element and constitutes several enzymes such as manganese superoxide dismutase that protect cells from damage induced by free radicals. When it is in excess, it causes poisoning, while its deficiencies affect carbohydrate and lipid metabolism [8]. Nickel is widespread in the environment, and it is an essential element for organisms. It becomes toxic at high levels, in particular, contributing to an increase of ROS (reactive oxygen species) formation, increasing oxidative damage at the cellular level, but it could be also genotoxic and immunotoxic [42]. In this work, the average concentration of nickel detected in fur is higher than that identified in red deer belonging to a northwest region of Italy [27]. This discrepancy could depend on the differences in the regional loads of this element. Selenium is a non-metal that is involved in a lot of biological functions and is a component of antioxidant enzymes such as glutathione peroxidase and thioredoxin reductase. Moreover, selenoenzymes are important for reducing oxidative damage in the brain and neuroendocrine tissues. At high concentrations, Se is very toxic. The mean concentration of selenium in the fur of roe deer detected in this research is higher than the levels detected in European bison hair [43]; it is, however, similar to the detected concentrations in camel and alpaca fur [28,44]. This variation could be explained by the different selenium levels in the plants at the base of the animals’ diets [45]. Zinc is one of the most important nutrients in human and animal nutrition, able to strongly interfere with health. Indeed, zinc is an essential element involved in a variety of biochemical processes, such as hair and skin growth, as it is involved in the building of keratin; moreover, a lot of enzymes, proteins, and transcription factors need to bind to this element to exert their functions [28]. The mean level of zinc detected in this study is similar to that detected in cows [37] but lower than those identified in deer and marmots [27]. The differences could depend on interspecific physiological differences, but they also could depend on factors related to the sampling area.

It is widely recognized that each geographical area owns specific elemental patterns. A lot of studies have already demonstrated that the metal availability in soils depends on the geochemistry, and usually, plants reflect the soil mineral profile. Bioaccumulation or biodilution may occur in the biological matrix, such as hair. Indeed, this matrix is influenced by several factors such as the physiology, diet, and metabolism of the animal species considered [46,47].

### 4.1. Comparison between Rural and Urbanized Areas

All wild animals, including roe deer, have a diet closely related to the natural environment. Due to anthropogenic activities, in some areas, soil results are polluted by considerable amounts of metals [24]. The transfer of elements from soil to biota and plants depends on soil conditions, such as the organic matter content, pH, redox potential, and clay content [48]. Because of this, wild roe deer have access to variable quantities of essential and toxic elements depending on their home range. In this study, statistically significant differences were detected in the fur content of iron, magnesium, manganese, aluminium, and chromium between roe deer from urbanized and rural areas. In particular, the content of elements resulted higher in the urbanized than in the rural area (Table 5). The other elements analysed showed no significant statistical differences, but in all cases, the mean concentration and the median values were higher in fur from roe deer belonging to the urbanized area. For decades, wild animals have been used as indicators of essential metals’ bioavailability or for assessing the presence of toxic elements due to their strong binding with the natural environment [24,49,50]. The results obtained in this study are in agreement and also support the studies conducted by other authors in which significant differences between the areas of origin of animals were detected [50]. The area of about 5000 ha in the study was divided into two zones, rural and urbanized, based on the number of disclosed anthropic activities. Despite the limited distance between the two zones, the statistical analysis showed significant differences between the animals sampled in the rural area and those sampled in the urbanized area. Thus, considering these results, it is possible to assume that due to the limited home range, the roe deer can reflect the conditions of a given area in a precise way, becoming a useful indicator of both environmental pollution and trace elements’ presence and their bioavailability.

### 4.2. Comparison between the Class of Age

It is widely recognized that different trace and toxic elements might be deposited in different tissues at different rates. Moreover, differences in deposition might not depend only on the intrinsic characteristics of the tissues; indeed, several studies have demonstrated that there might be large age-related variations [51]. The information about age-related variability in trace element concentrations in deer, roe deer, and other wild animals is scarce and fragmentary. In this study, Mg and Cr levels were found to be significantly higher in older animals. Many authors have investigated trace elements in different tissues of roe deer, for example, in antlers, bones, and soft tissues, identifying an age-related pattern of bioaccumulation [52,53]. The other elements under study did not show significant statistical differences; however, the average concentration and the median were higher in older animals. These results agree with what Garcia et al. reported in a study conducted on the liver, kidney, and muscle of roe deer; except for the zinc, they found higher concentrations in young animals, while we observed similar concentrations in the two age classes (Table 6) [54]. In this type of survey, the age of the animal must be considered for the correct interpretation of the presence of xenobiotics because their half-life is extremely long. As previously reported, several age-related factors could affect the response of sentinels to pollutants; among these, the most important is certainly the longer exposure time [52].

### 4.3. Comparison between Sexes

In the literature, it is widely reported that male and female organisms have significant differences in exposure, toxicokinetics, and responses to chemicals. In this study, the hair contents of copper, iron, magnesium, cadmium, and chromium significantly differed between males and females. In particular, copper, iron, magnesium, and chromium were higher in females than in males. Moreover, as shown in Table 7, for most elements, the concentration tended to be higher in the hair of females. The trend obtained in this study is consistent with the trend obtained in other studies, in which the concentration of potentially toxic elements and trace elements in muscle, kidney, liver, and fur were higher in roe deer does than in males [54,55]. The reasons for these differences could be explained by a gender-related biotransformation metabolic effect: males have higher MFO (mixed function oxidase) activity, enzymes that provide phase I metabolism, which makes xenobiotics easier to excrete. This function is enhanced by the presence of endogenous steroids such as testosterone [26]. Conversely from roe deer literature, in our study, the cadmium content was higher in the hair of females than in males [55]. However, in a study by French et al. [56], cadmium was found to be higher in female deer. Another variable to consider in the context of this study is that the hunting season, and thus the season of the year and the related climate, is different for males and females. Indeed, the diet of wild animals is strictly bound to the availability of nutrients in the natural environment. In the literature, it is widely reported that the season strongly influences the diet of wild animals and, consequently, the content of toxic and trace elements in their tissues [56]. Therefore, it is still unclear if the obtained results reflect the different physiology related to the sex of the animals or if they are due to the hunting season.

## 5. Conclusions

The concentrations of trace elements considered toxic vary widely between organs and animal species and in general, the toxicity levels found in hair cannot be compared with known levels found in other tissues and organs due to differences in distribution factors related to physiological characteristics (such as perfusion, composition, and metabolic rate). Environmental pollution due to toxic metals causes severe adverse effects on the health of humans and animals; therefore, hair from mammals may be a useful bioindicator of environmental pollution over a long time, thanks to the accumulation pattern shown by this matrix. In this study, hair was shown to be a good non-invasive bioindicator tool to assess the levels of potentially toxic elements (PTEs) in animals. Moreover, the levels of trace and toxic elements associated with anthropogenic activities showed variation between geographical areas, age, and sex. In conclusion, the hair of wild animals, in particular, those obtained from highly territorial animals such as roe deer, could be useful to indicate environmental contamination or hot spots. Further investigations comparing the fur/hair elements content between wildlife and livestock belonging to the same geographical area are warranted to explore the feasibility of this matrix from roe deer in reflecting toxic and trace elements accumulation in domestic ungulates sharing their graze zone.

## Figures and Tables

**Table 1:** ICP-OES device parameters for determination of trace and toxic elements.

Parameters	Assigned Value
***Plasma gas flow rate***	15 L/min
***Argon carrier flow rate***	0.5 L/min
***Sample flow rate***	1.51 L/min
***The speed of peristaltic pump***	100 rpm
***RF Power***	1150 W

**Table 2:** Wave lengths used in the analysis for each element.

Elements	Wave Length (nm)
*Aluminium (Al)*	167.070
*Arsenic (As)*	189.042
*Cadmium (Cd)*	228.802
*Chromium (Cr)*	267.716
*Copper (Cu)*	324.754
*Iron (Fe)*	259.940
*Magnesium (Mg)*	285.213
*Manganese (Mn)*	257.610
*Nickel (Ni)*	341.476
*Lead (Pb)*	220.353
*Selenium (Se)*	196.090
*Zinc (Zn)*	206.200

**Table 3:** The results of ICP-OES method validation for elements.

Elements	Quality Control (QC)	LOD *	LOQ *	Expected Concentration	Measured Concentration (*n* = 3) (ppm)	Precision (RSD%)
Cr	QC-1 QC-2	0.003	0.008	0.500 1.000	0.500 1.000	1.632 0.816
Cu	QC-1 QC-2	0.002	0.006	0.500 1.000	0.510 1.010	1.227 0.538
Fe	QC-1 QC-2	0.003	0.004	0.500 1.000	0.490 0.990	5.772 0.846
Al	QC-1 QC-2	0.001	0.003	2.500 5.000	2.420 5.000	0.168 0.144
Ni	QC-1 QC-2 QC-3 QC-4	0.000	0.002	0.050 0.100 0.500 1.000	0.050 0.100 0.490 0.990	2.817 1.694 0.838 0.124
Zn	QC-1 QC-2	0.003	0.009	0.500 1.000	0.540 1.000	0.756 0.343
Se	QC-1 QC-2	0.000	0.001	0.050 0.100	0.050 0.100	1.632 0.816
Mn	QC-1 QC-2	0.000	0.000	0.050 0.100	0.050 0.100	4.320 1.414
As	QC-1 QC-2 QC-3 QC-4	0.000	0.001	0.050 0.100 0.500 1.000	0.050 0.100 0.490 0.960	5.684 0.974 4.248 1.274
Mg	QC-1 QC-2	0.000	0.001	0.500 1.000	0.490 1.010	0.419 1.011
Pd	QC-1 QC-2	0.000	0.004	0.500 1.000	0.490 1.010	3.332 0.451
Cd	QC-1 QC-2	0.008	0.014	0.500 1.000	0.490 1.004	0.419 1.044

QC: quality control; LOD: limit of detection; LOQ: limit of quantitation; RSD: relative standard deviation. * The LOD and LOQ unit of Cr, Cu, Mn, Se, As, Cd, Ni and Pd is μg/g sample; The LOD and LOQ unit of Al, B, Fe, Mg and Zn is mg/g sample.

**Table 4:** Average concentrations of trace and toxic elements.

	Concentration (mg·kg^−1^)		
Element	Mean ± SD	Median	Minimum-Maximum
***Cr***	1.61 ± 1.04	1.31	0.60–6.58
***Cu***	7.49 ± 2.09	6.95	4.71–12.21
***Mn***	3.10 ± 2.40	2.46	0.64–9.71
***Se***	1.61 ± 1.23	1.58	0.09–6.15
***As***	1.97 ± 1.69	1.42	0.05–6.80
***Cd***	0.08 ± 0.16	0.05	0.00–0.99
***Ni***	0.82 ± 1.13	0.54	0.00–6.78
***Pb***	1.39 ± 1.63	0.99	0.01–9.42
***Al***	140 ± 280	60	20–1800
***Fe***	160 ± 144	110	30–670
***Mg***	230 ± 120	170	110–680
***Zn***	70 ± 10	70	50–90

**Table 5:** Differences detected between urbanized and rural areas (mg·kg^−1^).

Element		Urbanized	Rural
Cu	mean ± SD	7.81 ± 2.04	7.18 ± 1.48
	median	7.84	6.22
	minimum–maximum	4.71–12.21	4.91–11.40
Fe **	mean ± SD	220 ± 170	90 ± 60
	median	170	60
	minimum–maximum	30–670	50–200
Mg *	mean ± SD	260 ± 140	200 ± 100
	median	220	160
	minimum–maximum	140–680	110–490
Mn **	mean ± SD	4.35 ± 2.79	1.91 ± 1.05
	median	3.58	1.75
	minimum–maximum	1.12–9.71	0.64–4.21
Se	mean ± SD	1.70 ± 1.42	1.52 ± 1.06
	median	1.58	1.58
	minimum–maximum	0.09–6.15	0.22–3.15
Zn	mean ± SD	70 ± 10	70 ± 10
	median	70	60
	minimum–maximum	60–90	50–90
Al *	mean ± SD	220 ± 390	70 ± 60
	median	90	50
	minimum–maximum	30–1800	20–240
As	mean ± SD	2.26 ± 2.05	1.69 ± 1.24
	median	1.54	1.34
	minimum–maximum	0.10–6.79	0.05–4.59
Cd	mean ± SD	0.10 ± 0.22	0.06 ± 0.05
	median	0.05	0.04
	minimum–maximum	0.00–0.99	0.00–0.17
Cr ***	mean ± SD	2.01 ± 1.33	1.23 ± 0.43
	median	1.60	1.18
	minimum–maximum	0.94–6.58	2.22
Ni	mean ± SD	0.68 ± 0.58	0.96 ± 1.49
	median	0.60	0.53
	minimum–maximum	0.00–2.29	0.00–6.78
Pb *	mean ± SD	1.99 ± 2.09	0.83 ± 0.70
	median	1.81	0.60
	minimum–maximum	0.01–9.42	0.05–2.25

Difference between urbanized and non-urbanized areas = *: *p* < 0.05; **: *p* < 0.01; ***: *p* < 0.001.

**Table 6:** Differences detected between the classes of age (mg·kg^−1^).

Element		Aged < 36 months	Aged ≥ 36 months
Cu	mean ± SD	6.96 ± 1.61	7.90 ± 2.35
	median	6.16	7.93
	minimum–maximum	5.25–11.40	4.71–12.21
Fe	mean ± SD	120 ± 80	180 ± 170
	median	110	120
	minimum–maximum	30–330	30–670
Mg *	mean ± SD	180 ± 70	260 ± 140
	median	160	210
	minimum–maximum	110–360	120–680
Mn	mean ± SD	2.53 ± 1.57	3.54 ± 2.76
	median	2.32	2.65
	minimum–maximum	0.91–6.87	0.64–9.71
Se	mean ± SD	1.20 ± 0.85	1.93 ± 1.40
	median	1.16	1.99
	minimum–maximum	0.09–2.67	0.22–6.15
Zn	mean ± SD	70 ± 10	70 ± 110
	median	60	70
	minimum–maximum	60–90	50–90
Al	mean ± SD	86 ± 68	190 ± 170
	median	60	70
	minimum–maximum	20–240	20–180
As	mean ± SD	1.93 ± 1.33	2.00 ± 1.96
	median	1.42	1.33
	minimum–maximum	0.22–4.24	0.05–6.80
Cd	mean ± SD	0.067 ± 0.05	0.087 ± 0.20
	median	0.057	0.03
	minimum–maximum	0.00–0.17	0.00–0.99
Cr **	mean ± SD	1.17 ± 0.36	1.96 ± 1.26
	median	1.12	1.63
	minimum–maximum	0.06–1.86	0.81–6.58
Ni	mean ± SD	0.63 ± 0.57	0.97 ± 1.42
	median	0.57	0.43
	minimum–maximum	0.00–2.30	0.00–6.78
Pb	mean ± SD	1.08 ± 0.86	1.63 ± 2.02
	median	1.86	1.10
	minimum–maximum	0.05–2.74	0.01–9.42

Difference between animals aged < 36 months and ≥ 36 months = *: *p* < 0.05; **: *p* < 0.01.

**Table 7:** Differences detected between sexes (mg·kg^−1^).

Element		Female	Male
Cu *	mean ± SD	8.32 ± 2.16	6.85 ± 1.83
	median	8.39	6.12
	minimum–maximum	4.83–12.04	4.71–12.21
Fe *	mean ± SD	160 ± 80	150 ± 180
	median	170	60
	minimum–maximum	50–370	30–670
Mg *	mean ± SD	280 ± 150	180 ± 70
	median	270	170
	minimum–maximum	130–680	110–410
Mn	mean ± SD	2.86 ± 2.04	3.29 ± 2.68
	median	2.55	2.25
	minimum–maximum	0.79–9.71	0.64–9.21
Se	mean ± SD	1.98 ± 1.35	1.33 ± 1.08
	median	1.58	0.74
	minimum–maximum	0.30–6.15	0.09–3.06
Zn	mean ± SD	70 ± 10	70 ± 10
	median	70	60
	minimum–maximum	50–90	50–90
Al	mean ± SD	210 ± 410	90 ± 80
	median	90	60
	minimum–maximum	30–1800	20–330
As	mean ± SD	1.45 ± 1.31	2.37 ± 1.86
	median	1.13	1.63
	minimum–maximum	0.05–4.58	0.10–6.80
Cd *	mean ± SD	0.09 ± 0.24	0.07 ± 0.05
	median	0.03	0.06
	minimum–maximum	0.00–0.99	0.00–0.17
Cr ***	mean ± SD	2.13 ± 1.33	1.21 ± 0.49
	median	1.86	1.13
	minimum–maximum	1.00–6.58	0.60–2.55
Ni	mean ± SD	1.01 ± 1.62	0.68 ± 0.52
	median	0.51	0.56
	minimum–maximum	0.00–6.78	0.00–2.30
Pb	mean ± SD	1.80 ± 2.26	1.07 ± 0.81
	median	1.23	0.95
	minimum–maximum	0.01–9.42	0.05–2.48

Difference between female and male = *: *p* < 0.05; ***: *p* < 0.001.

## Data Availability

The data presented in this study are available on request from the corresponding author. The data are not publicly available due to privacy restrictions.
